# Editorial: Alcohol and drug use in low- and middle-income countries

**DOI:** 10.3389/fpsyt.2024.1381726

**Published:** 2024-02-19

**Authors:** Samer El Hayek, Victor Lasebikan, Alireza Noroozi

**Affiliations:** ^1^ Medical Department, Erada Center for Treatment and Rehabilitation in Dubai, Dubai, United Arab Emirates; ^2^ College of Medicine, University of Ibadan, Ibadan, Nigeria; ^3^ Iranian National Center for Addiction Studies (INCAS), Tehran University of Medical Sciences (TUMS), Tehran, Iran

**Keywords:** addiction, substance use disorder, alcohol, drugs, substance, LMICs

Rates of substance use disorders (SUDs) and their impact on physical and mental wellbeing have been rising in low- and middle-income countries (LMICs). Novel trends in the types and patterns of drug use are also emerging (1–4). Although a majority of consumers live in and purchase drugs from developed wealthy nations, production is conventionally linked to LMICs (1, 5). In that regard, knowledge and research on SUDs in LMICs, as well as national responses to prevent, treat, and decrease the harms of alcohol and drug use, remain limited. Common barriers to treatment include a perceived lack of problems and low motivation (6). Furthermore, gender inequality, poverty, illiteracy, disparities in access and receipt of primary prevention, treatment, and harm reduction services, and scarcity of resources and professionals working in addiction medicine in LMICs have negatively affected the effectiveness, establishment, longevity, and quality of services (7, 8).

In this Research Topic titled “*Alcohol and Drug Use in Low- and Middle-Income Countries*”, we examine some of the latest data on alcohol and substance use in LMICs. The included studies mainly emanate from Ethiopia, Iran, Honduras, and Pakistan. The findings highlighted in this Research Topic represent merely the tip of the iceberg when it comes to the characteristics, impact, and treatment of SUDs in LMICs. Our Research Topic underscores the urgent need for further studies across LMICs to capture the magnitude of alcohol and drug use in these societies.

In Ethiopia, Kassew et al. assessed the prevalence of substance use among teenagers and the individual- and community-level characteristics that might influence it. The survey included 10,594 individuals aged 15 to 24 years. Past-month prevalence of substance use reached 46.74%. Of the participants, 36.34%, 12.56%, and 0.95% were drinking alcohol, chewing Khat, and smoking tobacco products, respectively. Factors significantly associated with alcohol or substance use included male sex, age 20 to 24 years, exposure to media, having an occupation, and living in an urban area. The authors suggested multiple interventions targeting this population, including banning mass-media alcohol advertising, restricting alcohol, Khat, and tobacco product marketing to minors, and prohibiting smoking in public places. On the other hand, Kassew et al. looked at psychotic symptoms among youth using psychoactive substances in Ethiopia. Of 372 participants, 79.57%, 53.49%, 34.14%, and 16.13% reported using alcohol, Khat, tobacco/cigarette products, and other substances (inhalants and illicit drugs). The prevalence of psychotic symptoms was 24.2%. Factors significantly associated with psychotic symptoms included being married, recently losing a loved one, having low perceived social support, and having severe psychological distress. Findings highlighted the importance of diligent screening for psychotic symptoms, particularly among those with limited support and increased psychological distress.

Two studies emanating from Iran particularly looked at individuals with opioid use disorder. Noroozi and Danesh investigated the prevalence of alcohol use among 706 individuals receiving opioid agonist treatment from different certified centers in Northern Iran. The prevalence of lifetime and past-month history of alcohol consumption was estimated at 39.2% and 6.9%, respectively. The prevalence of a history of excessive alcohol use on one occasion reached 18.8%. The estimated past-month prevalence of alcohol use was lower than the reported prevalence in countries where the production, distribution, and consumption of alcohol are legal. The authors concluded that alcohol consumption among patients receiving opioid agonist treatment is common, despite a total ban on alcohol consumption in Iran.

In a retrospective cohort study of 105 male participants receiving methadone for opioid use disorder, Radfar et al. compared treatment retention rate between the people referred from compulsory residential centers (56% of participants) and volunteer patients. Although the average treatment adherence time for non-referred voluntary patients was about 60 days higher than those referred from compulsory residential centers, the authors found no significant differences in retention days and one-year retention rate between groups. The total one-year retention rate was 15.84%: 12.28% in those referred from compulsory residential centers and 20.45% in non-referred voluntary patients. Among the studied variables, only marital status was significantly associated with retention. The authors suggested the need for larger sample sizes and longer follow-ups to understand the efficacy of compulsory treatment.

Shifting gears, Espinoza-Turcios et al. looked at the mental health of the Honduran population during the COVID-19 pandemic using the Beck Hopelessness Scale, and Hamilton scales for depression and anxiety. Of 8,125 participants, 14.9%, 12.9%, and 1.2% screened positive for hopelessness, depression, and anxiety, respectively. Of the significantly associated factors, consuming drugs in the last 6 months was associated with more hopelessness and depression.

Lastly, in Pakistan, Nawaz et al. assessed the magnitude and associated factors of prescription drug dependence (PDD), in comparison to concomitant prescription drug dependence and illicit drug use (PIDU), in individuals attending three addiction treatment centers. Of 537 participants (93.3% males with an average age of 31 years), one-third (33.3%) met criteria for PDD. Of those, 71.9% reported benzodiazepines as their most frequently used drug, followed by narcotic analgesics (56.8%), cannabis/marijuana (45.5%), and heroin (41.5%). Compared to PIDU, PDD was significantly less associated with the injectable route of use and psychotic symptoms. Reported reasons for PDD included pain, low mood, and insomnia. PDD was significantly associated with an attitude that prescription drugs are safer than illicit drugs and patients reported alprazolam, buprenorphine, nalbuphine, and pentazocin use as alternatives to illicit drugs. The authors highlighted the implications of these findings on intervention strategies that particularly target PDD.

In conclusion, the studies included in this Research Topic provide valuable insights into the prevalence, characteristics, and associated factors of SUDs in diverse LMICs. Noteworthy findings include the prevalence of SUDs in youth in Ethiopia and potential co-occurring psychotic symptoms, the common occurrence of alcohol consumption among individuals receiving opioid agonist treatment in Iran, as well as the complex interplay between referral source and treatment adherence. The impact of drug consumption on mental health during the COVID-19 pandemic is well-documented in Honduras. Moreover, the assessment of prescription drug dependence in Pakistan sheds light on the prevalence and associated factors, emphasizing the need for targeted intervention strategies.

Despite these contributions, it is evident that there remains a substantial gap in research on SUDs in LMICs. To address this gap and improve our understanding of the magnitude and nuances of SUDs in LMICs, it is imperative to encourage future national and regional research endeavors. Collaboration between scientists, healthcare workers in the field of addiction, institutions, and policymakers is crucial for developing comprehensive and culturally sensitive interventions. Furthermore, larger sample sizes, longer follow-up periods, and holistic multidisciplinary approaches can enhance both research and clinical outcomes. Prioritizing further investigation into the prevalence, characteristics, and impacts of SUDs in LMICs will inform evidence-based interventions, promote public health, and mitigate the global burden of alcohol and drug use.

## Author contributions

SH: Writing – original draft, Writing – review & editing. VL: Writing – review & editing. AN: Writing – review & editing.
